# Silencing of LINE-1 retrotransposons contributes to variation in small noncoding RNA expression in human cancer cells

**DOI:** 10.18632/oncotarget.1822

**Published:** 2014-03-22

**Authors:** Stephen Ohms, Danny Rangasamy

**Affiliations:** ^1^ John Curtin School of Medical Research, The Australian National University, Canberra, Australian Capital Territory, Australia

**Keywords:** MicroRNAs, small RNAs, PIWI-interacting RNAs, Retrotransposons, Genome defense, Breast Cancer Cells.

## Abstract

Noncoding RNAs are key players in the maintenance of genomic integrity, particularly in silencing the expression of repetitive elements, some of which are retrotransposable and capable of causing genomic instability. Recent computational studies suggest an association between L1 expression and the generation of small RNAs. However, whether L1 expression has a role in the activation of small RNA expression has yet to be determined experimentally.; Here we report a global analysis of small RNAs in deep sequencing from L1-active and L1-silenced breast cancer cells. We found that cells in which L1 expression was silenced exhibited greatly increased expression of a number of miRNAs and in particular, members of the let-7 family. In addition, we found differential expression of a few piRNAs that might potentially regulate gene expression. We also report the identification of several repeat RNAs against LTRs, LINEs and SINE elements. Although most of the repeat RNAs mapped to L1 elements, in general we found no significant differences in the expression levels of repeat RNAs in the presence or absence of L1 expression except for a few RNAs targeting subclasses of L1 elements. These differentially expressed small RNAs may function in human genome defence responses.

## INTRODUCTION

Small RNAs have recently emerged as being important in the control of gene expression in almost all biological processes. Many different types of small RNA, including microRNAs (miRNAs), endogenous siRNAs (endo-siRNAs), small nucleolar RNAs (snoRNAs) and more recently, Piwi-interacting RNAs (piRNAs), are expressed in a variety of tissues and exert diverse effects on cellular functions [1-3]. The different classes of small RNAs are generally distinguished by their mode of biogenesis, protein partners and/or biological function [4]. miRNAs are generated from a single cleavage event of a short hairpin pre-miRNA by the Dicer enzyme, and are loaded onto Argonaute proteins and then guide translational repression of mRNAs. Some miRNAs can also promote degradation of mRNAs by deadenylation [5]. Endo-siRNAs are a class of small interfering RNA with sequences that are complementary to other endogenous mRNAs [6]. They are often generated from multiple Dicer cleavages of long precursor dsRNAs or generated from genomic sources that include the folding back of inverted repeats, sites of bidirectional transcription, and read-through antisense transcripts of retrotransposons and pseudogenes [7, 8]. Recent advances in high-throughput small RNA sequencing have resulted in the identification of a growing number of small RNAs in animals. These include many repeat-associated siRNAs (originally termed rasiRNAs), which are produced from retrotransposon-derived dsRNAs in *Drosophila* germline cells. These repeat-associated siRNAs are now known as piRNAs [9], since they are bound by Piwi proteins and are produced by a Dicer-independent mechanism. piRNAs have been shown to play a critical role in silencing retrotransposons as well as having a role in the assembly of heterochromatin structures to silence gene expression [10, 11].

The human genome contains large numbers of repetitive sequences and retrotransposable elements both within and between genes. These repeat elements, accounting for nearly 45% of the genome, are grouped into four classes: LINEs (long interspersed elements), SINEs (short interspersed elements), LTRs (long terminal repeats) and DNA transposons. The most active retrotransposons are the LINE-1 or L1 elements [12] which are capable of replicating through their own reverse transcriptase and endonuclease enzymes and inserting DNA copies at new genomic locations. This can often lead to deleterious effects in the genome, such as through the insertion of L1 DNA copies into protein-coding regions of genes, abolishing their function. They may also negatively affect genome integrity because of their ability to create dsDNA breaks during retrotransposition [13]. A recent study surveying human tumors and comparing their genomes to those of adjacent normal tissues suggested that tumors have high frequencies of L1 insertions and dsDNA breaks that are not present in normal tissues [14, 15]. The activation of L1 elements has also been implicated in the genomic insertions of ~8000 processed pseudogenes [16] and the retrotransposition of nonautonomous elements, such as retrogenes, Alu and SVA elements [17], which can affect genomic structure in a multitude of ways. Thus, to protect genome integrity, cells normally employ a number of defense strategies, with epigenetic mechanisms, such as silencing by small RNAs, DNA methylation, or histone modifications, playing key roles in keeping L1 elements in check.

Several studies carried out in somatic cells of *Drosophila* and in mouse oocytes have shown that endo-siRNAs regulate retrotransposons and protein-coding genes that are complementary to the endo-siRNAs [6, 18], similar to the function of piRNAs in germline cells [10]. Interestingly, recent evidence shows that piRNAs are also expressed in human cancer cells and their altered expression plays a role in the development of cancer [19]. In relation to this, we recently identified a subset of L1-specific siRNAs that are differentially expressed in a wide range of breast cancer cells compared to normal cells [20]. Overexpression of these siRNAs markedly silenced endogenous L1 expression through increased DNA methylation of the L1 promoter. These findings reveal a role for small RNAs in humans and indicate that depletion of L1-specific siRNAs is most likely to be one of the reasons for the activation of L1 elements in cancer cells. However, the expression status and roles of other small RNAs such as miRNAs and piRNAs have largely remained unknown. Several studies performed in human cancer cells have reported that blocking the retrotransposition of L1 elements not only controls L1 activity but also alters the expression of many genes involved in the proliferation and differentiation of cancer cells [21]. This raises an important question of how the silencing of L1 activity contributes to the regulation of these genes.

Nearly 25% of human promoters have been reported to contain retrotransposon-derived sequences including many *cis*-regulatory sequences [22], which are involved in specific patterns of gene expression. Interestingly, a significant number of miRNAs and other small RNAs are also derived from repetitive sequences [23, 24], which may in turn regulate the expression of other cellular genes harboring repetitive sequences in their transcripts. Through computational searches, two recent studies identified a number of small RNAs, including miRNAs and snoRNAs [25, 26], which are derived from the retrotransposition activity of L1 elements. Many of these small RNAs are surrounded by sequence features that are typical of L1 elements, such as target site duplications (TSDs) and poly-A tails at their 3' ends. These analyses propose the involvement of L1 retrotransposition in generating large numbers of small RNAs in the human genome [27]. Moreover, these studies also suggest the existence of many tissue-specific retrotransposon-derived small RNAs that may be a driving force for creating new regulatory elements involved in the control of gene expression [25, 26]. Despite these extensive bioinformatic analyses, the existence of such a mechanism needs to be experimentally verified. In light of the accumulating data suggesting a connection between L1 elements and the generation of small RNAs, we investigated this relationship using high-throughput deep sequencing. To investigate whether the expression of L1 elements was correlated with small RNA expression, we analyzed the profile of small RNA expression with respect to the activation or silencing of L1 elements using a published L1-specific endogenous siRNA sequence that specifically silences L1 expression in human cells [20]. Here, we show that rather than generating small RNAs, L1 expression globally reduces the expression of small RNAs. Conversely, silencing of L1 expression up-regulates the expression of a number of miRNAs and piRNAs, sometimes very markedly.

## RESULTS

### Generation of L1 silencing in breast cancer cells

**Figure 1:**
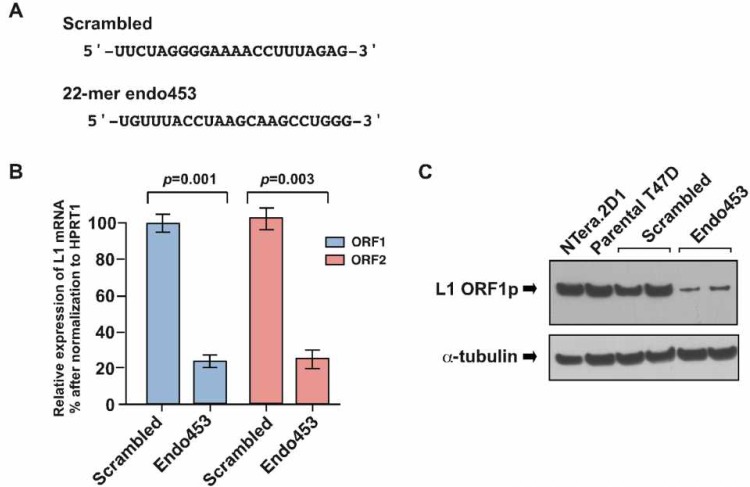
Endo453 sequence controls L1 expression (A) Sequences of L1-specific endo453 and scrambled control siRNAs are shown. (B) Quantitative RT-PCR analysis of L1 ORF1 and ORF2 mRNAs after stable transfection with endo453 and scrambled control siRNAs. Each bar represents the relative expression changes between L1-active (scrambled) and L1-silenced (endo453) cells after being normalized to HPRT1 as an internal control. Fold change was calculated from 2-ΔΔCT and p values by an unpaired t test. Error bars show s.d. (n=4). (C) Western blot of L1 protein in T47D cells stably transfected with endo453 and scrambled siRNAs. α-tubulin was used as a loading control. The whole cell extracts from NTera.2D1 and parental T47D cells were performed in parallel.

To determine whether the expression of L1 elements in human cells plays a role in controlling the level of small RNA expression, we used a human T47D breast cancer cell line because our recent studies showed high levels of L1 retrotransposition and concomitantly low levels of L1-specific siRNAs in these cells [15, 20]. To silence L1 expression we used a published L1-specific siRNA sequence (endo453) [20], that can specifically silence endogenous L1 element expression by targeting the L1 5'-UTR promoter region (at nucleotide positions 453 to 475). In this assay, we stably transfected T47D cells with an shRNA construct (mimicking the L1-specific endo453 sequence) whose expression was driven by constitutively expressing the U6 promoter. A scrambled endo-siRNA sequence with no homology to any known genes was used as a control (Figure 1A). As assessed by qRT-PCR analysis, we achieved L1 silencing efficiencies of 72 ± 4.6% (*p*=0.001) and 78 ± 5.8% (*p*=0.003) for ORF1 and ORF2 mRNA, respectively (Figure 1B). The effectiveness of the endo453 sequence was further confirmed by western blotting of whole-cell lysates from the endo453, scrambled or parental cells using anti-ORF1 antibodies (Figure 1C). Human embryonic carcinoma NTera.2D1 cells, which express high levels of L1 protein, served as a control. The resulting assays confirmed that the relative expression of L1 mRNA and protein was significantly reduced in endo453 cells compared with scrambled or the parental T47D cells. Together, these results show that the endogenous expression of human L1 elements can be efficiently silenced by an artificially transfected L1-specific siRNA sequence.

### Deep sequencing of small RNAs

To investigate whether the activation or silencing of L1 elements plays any role in contributing to small RNA expression, we used the Illumina GAIIx high-throughput sequencing platform to sequence small RNA libraries from the endo453 (L1-silenced) and scrambled (L1-active) cells. Since L1 expression was controlled by the introduction of exogenous (synthetic) endo453, the introduced sequences were bioinformatically removed from the files of Illumina reads prior to mapping in order to identify unbiased expression of small RNAs in the L1 silenced library. We then used the miRanalyzer small RNA mapping pipeline [28] to map the reads to miRBase and to identify and annotate all species of miRNAs present in the libraries [29]. In order to identify expressed piRNA sequences, we aligned the reads to the piRNABank database (human.tar.gz) [30] using CLC Genomics Workbench (version 5.5.1) (http://www.cicbio.com). Finally, we mapped the reads to the deepBase repeat-associated small RNA database (Human_sense_rasRNA_deepBase.fasta, Human_antisense_rasRNA_deepBase.fasta) [31] to detect repeat-associated RNA also using CLC Genomics Workbench. An overview of the sequencing and mapping results for the two libraries is given in Table 1.

Table 1Distribution of small RNAs in L1-active and L1-silenced libraries

**Table:** a) Alignment against microRNAs (output from alignment with the miRanalyzer pipeline)

	Mature		Ambig. mature		Mature-star		Ambig. Mature-star		Hairpin		Ambig. Hairpin	
	L1-active	L1-silenced	L1-active	L1-silenced	L1-active	L1-silenced	L1-active	L1-silenced	L1-active	L1-silenced	L1-active	L1-silenced
Number of known miRNAs detected	407	460	46	55	101	110	2	1	306	339	53	61
Fraction of known miRNAs^a^	39.4% (1032)	44.6% (1032)	---	---	0.0% (191)	0.0% (191)	---	---	29.2% (1048)	32.3% (1048)	---	---
Unique reads	16156	18913	438	536	933	1016	3	1	1875	2467	434	449
Fraction of unique reads^b^	3.10%	4.10%	0.08%	0.12%	0.18%	0.22%	0.00%	0.00%	0.36%	0.54%	0.08%	0.10%
Read count^c^	3882279	8076212	17555	29845	33175	41642	17	10	51443	162308	33067	37910
Fraction of total read count^d^	18.30%	35.50%	0.08%	0.13%	0.16%	0.18%	0.00%	0.00%	0.24%	0.71%	0.16%	0.17%

a fraction of known miRNAs: % of known microRNAs detected in the sample/total number of known microRNAs in miRBase

b fraction of unique reads: the fraction of unique reads in the library that mapped to miRBase

c read count: the total count of all reads mapped to mirBase (i.e. the sum over all unique reads in the library of the number of times each unique read appears)

d fraction of total read count: read count in c. above as a percentage of the total number of reads in the library (21,266,358 for L1-active and 22,745,765 for L1-silenced as described in Results)

**Table:** b) Alignment against Piwi-interacting RNAs (output from alignment against human piRNABase)

	hsa_piRNAs	
	L1-active	L1-silenced
Number of known piRNAs detected	314	340
Fraction of known piRNAs detected	1.34%	1.45%
Read count	77732	70869

**Table:** c) Alignment against repeat RNAs (output from alignment against deepBase human rasRNA)

	Antisense		Sense		Ratio of antisense/sense	
	L1-active	L1-silenced	L1-active	L1-silenced	L1-active	L1-silenced
LINE	141597	103436	137990	83348	1.02	1.24
SINE	26351	36022	34337	33628	0.7	1.07
LTR	47552	43448	34073	24638	1.39	1.76
DNA_transposon	15165	15052	4050	4614	3.74	3.26
Satellite DNA	428	388	104	117	4.11	3.31
Small cytoplasmic RNA	0	0	9045	30041		
Unknown class/family	4866	6299	220374	307964	0.02	0.02
Unknown genomic repeat	309	233	104	117	2.97	1.99
rRNA	64	22	61071	75260	0.001	0.0002

**Figure 2:**
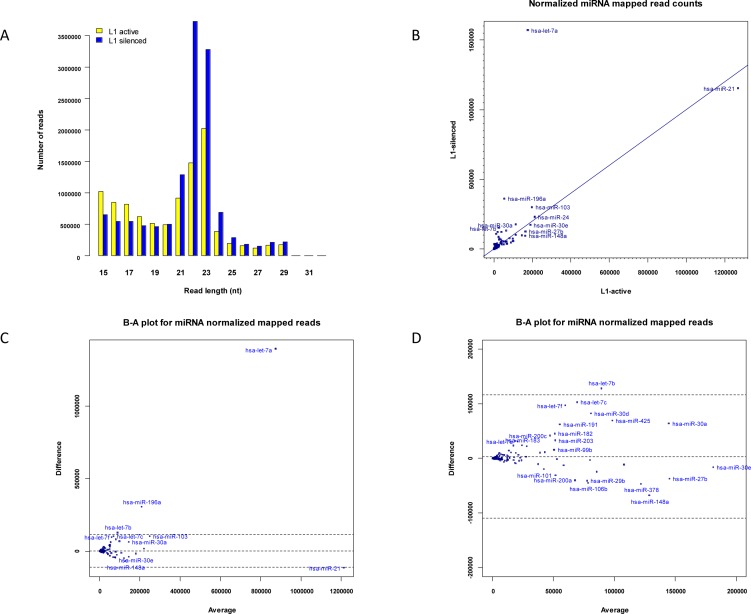
Expression of miRNAs (a) Length distribution of small RNAs in L1- active and L1-silenced cells. (b) Scatter plot showing normalized read counts for miRNAs in L1-silenced cells versus L1-active. Blue symbols denote miRNAs. (c) Bland-Altman plot of normalized read counts for miRNAs in L1-silenced library versus L1-active. The difference in the read counts (L1-silenced – L1-active) (vertical axis) is plotted against the average of the read counts [(L1-silenced + L1-active)/2]. If the read count for a particular miRNA species is identical in both libraries, the point is located on the middle horizontal line. The other horizontal lines represent the ± 2SD thresholds for the data. Points lying further away from the middle horizontal line are more likely to be significantly differentially expressed. (d) Higher magnification view of left-hand side of Figure 2c.

The distribution of lengths of the L1-silenced small RNAs was similar to that of the L1-active library, with peaks at 22–23-nt (Figure 2A). After removal of singleton reads and reads of less than 17-nt from each library by the miRanalyzer pipeline, there were 21,266,358 and 22,745,765 reads in the L1-active and L1-silenced libraries, respectively. Surprisingly however, 8,076,212 sequence reads from the L1-silenced sample mapped to known mature miRNAs compared to 3,882,279 in the L1-active sample. This large increase in numbers of reads mapping to known miRNAs in the L1-silenced library was unexpected. However, we found that a small number of very highly differentially expressed miRNAs were responsible for most of the discrepancy (Figure 2C). In particular, in the intermediate miRanalyzer file of mapped reads at the step prior to normalization (by default named as microRNA.diffexpr in the miRanalyzer pipeline), 1,855,633 reads mapped to *let-7a* in the L1-silenced library compared to 149,428 reads in the L1-active library, accounting for ~41% of the difference in the unnormalized counts of the mapped sequence reads. This large difference remained even after normalizing the read counts in both libraries using the DESeq package which is incorporated into the standard miRanalyzer pipeline [32]. DESeq uses a median-based scaling method to normalize read counts rather than scaling based on the total read counts in each library. The key steps are as follows: first a reference sample is constructed by calculating the geometric mean over all samples of the counts for each mapped miRNA species. Next, for each sample, the quotient of the mapped read count of each miRNA species to that in the reference sample is calculated. The quotients for each sample are sorted in numerical order and the median quotient becomes the scaling factor for the sample. This is a robust method of normalizing read counts when a relatively few highly differentially expressed small RNA species might bias the normalization of the library sizes if a global scaling method based on total read counts in each library was used [32, 33].

### Differentially expressed miRNAs

To identify miRNAs that were differentially expressed between active and L1-silenced cells, we mapped the reads from both libraries against miRBase (version 16) using the miRanalyzer pipeline [29], which also includes normalization and calculation of fold changes in the R/Bioconductor DESeq software package (version 1.6.0) [32]. To determine the rankings of differentially expressed miRNAs, we used the GFOLD (generalized fold change) algorithm (version 1.0.7) [34]. The GFOLD algorithm takes into account the observation that the fold changes of expressed species with low read counts are less reliable than those of species with high read counts and estimates a fold change value for each species based on the posterior distribution of the raw fold change. The analysis revealed numerous differentially expressed and highly expressing miRNAs in the L1-silenced versus the L1-active cells (Supplementary Table S1). Overall, 23 miRNAs were up-regulated in the L1-silenced cells based on a GFOLD (0.01) threshold of 2. The most abundantly expressed miRNAs were the let*-7* family members; *let-7a, let-7b, let-7c, let-7e* and *let-7f* were all up-regulated in the L1-silenced cells, in some cases, very markedly (Figure 2C). This result is consistent with published miRNA-profiling studies in many human cancer cells in which down-regulation of *let-7* family members has been reported more commonly than their up-regulation [35]. In addition to the let-7 family members, large changes were also seen in the expression of miR-196a, miR-30d, miR-103, miR-425, miR-191 and miR-200c (Figure 2D). Although these miRNAs were expressed at high levels in both libraries, the differences in their expression between the two libraries helped to account for most of the difference in the total number of mapped reads between the two libraries.

The canonical miRNA biogenesis pathway generates an miRNA duplex that consists of an miRNA and miRNA* strand. Several miRNA* species have been recently found in humans and some of them have beenimplicated in differentiation of cancer cell states [36]. In the current study, we identified three typical miRNA* species (miR-149*, miR-625* and miR-9*) that were exclusively expressed in the L1- silenced cells with normalized read counts ≥ 10 (Supplementary Table S1). In addition, we found three miRNA*s (miR-664*, miR-505* and miR-32*) that were differentially expressed between the two cell types with at least a 5x fold change. Several studies have described miRNA biogenesis by an alternative miRNA-processing pathway that uses the intron-splicing machinery instead of the Drosha/Dgcr8 endonuclease to generate miRNA precursors (called miRtrons) from intronic sequences [37]. One of the most highly differentially expressed was miR-664, a typical miRtron whose miRNA-664 (4 vs. 31) and miR-664* (25 vs. 346) sequences flank the edges of its host intron in a pattern reminiscent of miRtron miRNA precursors. The host gene of the miR-664 intron has been identified as RAB3GAP1, and the sequence of miR-664 overlaps with an annotated snoRNA, ACA38B [27]. PhastCons scores, which reflect probabilities of selective maintenance through evolution, extend to the edges of the miRNA hairpin (data not shown), suggesting that the miR-664 locus is highly conserved in the human, chimpanzee and mouse lineages. Moreover, we also found a number of newly identified miRNAs that were expressed only in the L1-silenced cells although at low levels, including miR-3662, miR-3174, miR-3146, miR-3192 and miR-3605-5p (Supplementary Table S1).

**Figure 3:**
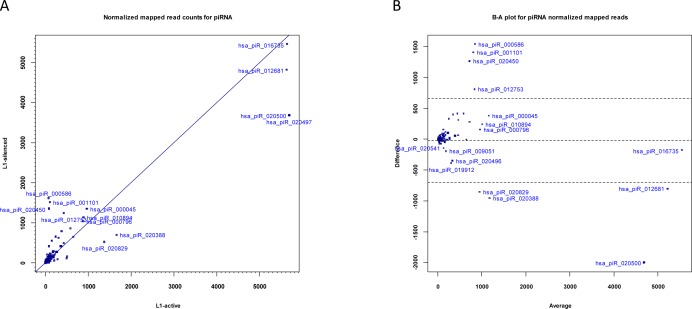
Expression of piRNAs (a) Scatter plot showing normalized read counts for piRNAs in L1-silenced cells versus L1-active. Blue symbols denote piRNAs. (b) Bland-Altman plot of normalized read counts for piRNAs in L1-silenced cells versus L1-active.

### Differential expression of piRNAs

piRNAs are a distinct class of small RNAs that interact with Piwi proteins and are involved in epigenetic and post-transcriptional silencing of retrotransposons and other genomic repeats in germline cells. Unlike miRNAs, which are in the size range of 21-23 nt, piRNAs are 24-31 nt in length. piRNA sequences do not have a precursor with a stem-loop and are not processed by Dicer. While there have been few reports of the presence of Piwi proteins in human cancers [38], one recent study did confirm the expression of piRNAs in human gastric cancer cells [39]. To identify differentially expressed piRNAs and other small RNAs in addition to miRNAs, we selected the adapter-trimmed reads in each library with lengths greater than 23-nt using the filter function in CLC Genomics Workbench and mapped this subset of reads from each library against the human piRNABank database [30]. After normalization of the reads with DESeq, a total of 77,732 reads in the L1-active and 70,869 reads in the L1-silenced cells matched 314 and 340 piRNA sequences, respectively, after deleting with a normalized mapped read count of 1 (Table 1). We found that the expression levels of most piRNAs were relatively constant in the L1-active and silenced libraries. However, we found a number of differentially expressed piRNAs in the L1-silenced versus the L1-active cells (Figure 3A). Overall, 22 and 26 piRNAs were up- and down-regulated, respectively in the L1-silenced cells based on the GFOLD thresholds of 2.0 and -0.5 respectively (Supplementary Table S2).

The most highly differentially expressed piRNAs as assessed by GFOLD were hsa_piR_000586 (fold change = 22x), hsa_piR_001101 (14x) and hsa_piR_020450 (16x) which were all up-regulated in the L1-silenced cells (Figure 3B). The extent of down-regulation of piRNA expression in the L1-silenced cells was not as marked as that of up-regulation. For instance, the most down-regulated piRNA in the L1-silenced cells as assessed by GFOLD was hsa_piR_020582 (fold change = −10.35x). Intriguingly, an NCBI BLAST search with the hsa_piR_000586 DNA sequence (28-nt) showed a match with 100% identity to a C/D box snoRNA residing in an intronic region of the Mortalin/GRP75 gene, an oncogene that is overexpressed in human cancers [40]. The E-value was 8×10^−7^. BLAST searches with the sequences of hsa_piR_001101 and hsa_piR_020450 also gave hits to C/D box snoRNAs residing in the introns of genes. These observations suggest that such piRNAs may have a role influencing gene expression. Although the functions of piRNAs are quite diverse and largely unknown, some of the differentially expressed piRNAs observed here may be involved in regulating cell activities and further studies in this area should reveal the functions of these piRNAs.

### Differential expression of repetitive RNA

Repeat-associated RNA refers to a class of noncoding small RNAs that includes transcripts from genomic repeats. Although it is unclear how they are processed and their biological functions are largely unknown, repeat-associated small RNAs map to repetitive elements such as LINEs, SINEs and LTRs. Like siRNAs, most repeat-associated small RNA sequences are in the size range of 18-23-nt. Until recently, all repeat-associated small RNAs were grouped together based on their genomic locations as rasRNAs [31]. While a number of earlier studies dismissed repeat-associated small RNA expression as genomic noise, one recent study identified functions for repeat RNA in the cell cycle and in DNA repair [41]. To identify differentially expressed repeat-associated small RNAs in addition to miRNAs and piRNAs, we mapped subsets of the small RNA libraries (i.e. reads < 24 nt in length from both the L1-active and L1-silenced libraries) against the human deepBase database [31]. The deepBase database contains human repeat RNAs that map to known repeat elements as well as large numbers of reads mapped to unique loci in the human genome. Reads were mapped against the human rasRNA antisense and sense fasta files downloaded from deepBase (Human_antisense_ rasRNA_deepBase.fasta and Human_ sense_rasRNA_deepBase.fasta as of July 2011). As with mapping against miRBase and piRNABank, the mapped read counts of each class of repeat RNA were normalized using the R/Bioconductor DESeq package [32]. The mapping procedure identified high levels of expression from many distinct repeat loci in both libraries. We analyzed the reads mapping to repeat RNA by first considering reads mapping to specific genomic loci, followed by grouping reads based on their repeat family and class.

**Figure 4:**
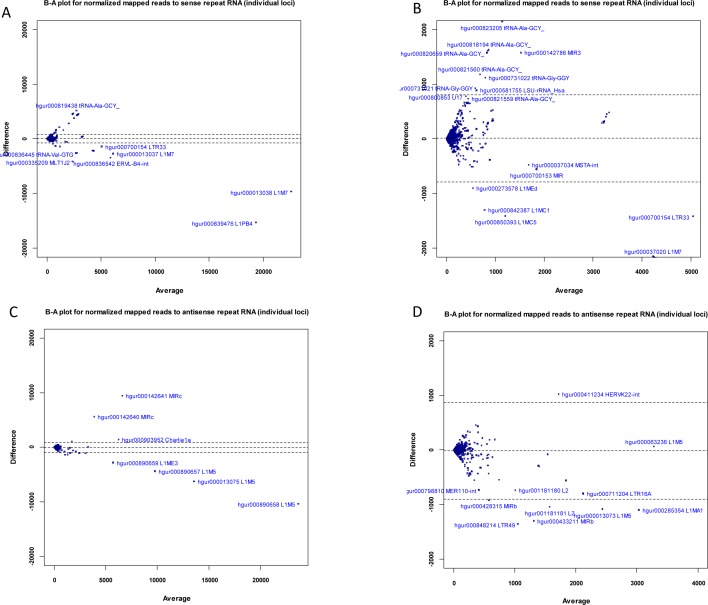
Expression of sense and antisense repeat-associated small RNAs (a) Bland-Altman plot of normalized read counts (mapped to individual genomic loci) for sense repeat RNAs in L1-silenced cells versus L1-active. (b) Higher magnification view of left-hand side of Fig4a. (c) Bland-Altman plot of normalized read counts (mapped to individual genomic loci) for antisense repeat RNAs in L1-silenced cells versus L1-active. (d) Higher magnification view of left-hand side of Fig4c. Blue symbols represent repeat-associated small RNAs.

Using Unix command-line tools and Perl and R scripts (available on request), we extracted the RepeatMasker [42] annotation of each mapped deepBase read (contained in the header of each read in the deepBase fasta files) and grouped the mapped reads by RepeatMasker class/family name. Read counts belonging to each class and family were summed up with R scripts to obtain the total reads belonging to each class and family of repeat element. After grouping reads into their repeat class (LINE, SINE etc), we found that for the LINE class, sense and antisense expression were very similar in the L1-active cells (ratio: 1.02, Table 1c), while in L1-silenced cells, LINE antisense expression was 24% of that of sense expression. At present, the relevance of this discrepancy is unclear but it is likely to be a result of L1 silencing. At the level of individual genomic loci (i.e., after annotating reads with their deepBase accession), we found differentially expressed sense and antisense loci. The Bland-Altman plots (Figure 4A-B) show that the sense loci most likely to be significantly down-regulated in the L1-silenced cells are hgur000839476 (belonging to the L1PB4 family) and hgur000013038 (belonging to the L1M7 family). There were also numerous differentially expressed loci with low levels of expression including repeats from the L1M7, L1MC5, L1MEd, L1MC1, LTR33 and ERVL-B4-int families (Figure 4A-B). For reads mapping to individual antisense loci, the loci most likely to be significantly down-regulated in the L1-silenced cells include hgur000890658, hgur000013075 and hgur000890657 (all belonging to L1M5) while hgur000142641 and hgur000142640 (belonging to MIRc) are up-regulated in L1-silenced cells (Figure 4C-D).

**Figure 5:**
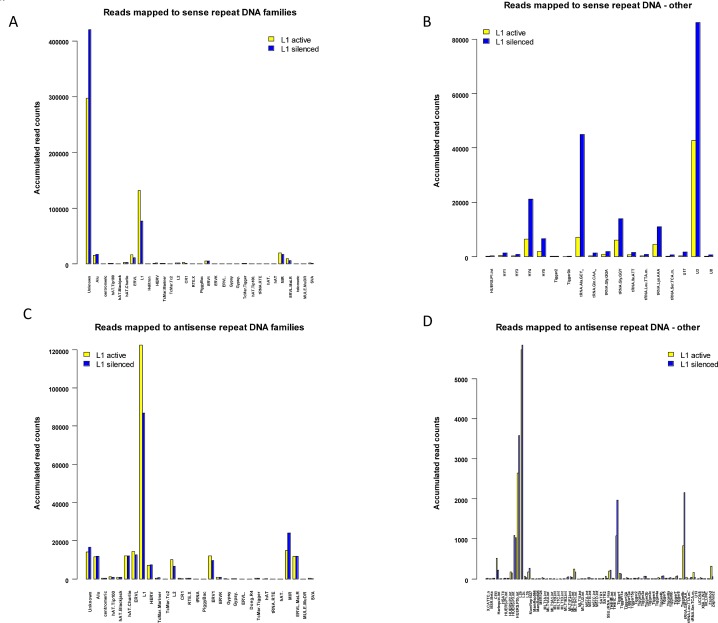
Mapping of sense and antisense reads to RepeatMasker families (a) Barplot showing accumulated normalized read counts of sense repeat-associated small RNAs after mapping L1-active and L1-silenced libraries against human deepBase sense rasRNA. The mapped reads were annotated with their RepeatMasker name and grouped into their RepeatMasker families. The totals of the normalized read counts belonging to each class are shown. Colour bars: L1-active sense reads, yellow: L1-silenced sense reads, blue. (b) Barplot showing a breakdown of the sense reads in the “Unknown” group. Only reads with a total accumulated count of greater than 10 in both libraries and a fold change greater than 2 are shown. Colour bars: L1-active sense reads, yellow: L1-silenced sense reads, blue. (c) Barplot showing accumulated normalized read counts of antisense repeat-associated small RNAs after mapping L1-active and L1-silenced libraries against human deepBase antisense rasRNA. (d) Barplot showing a breakdown of the antisense reads in the “Unknown” group.

The number of sense and antisense reads in each repeat RNA family and their relative fold changes between the two libraries are summarized in Figure 5A-D. In some cases, there was no RepeatMasker name for a particular deepBase entry and these reads are listed in the “Unknown” category and breakdowns of these are shown in Figures 5B and 5D. When grouped according to their RepeatMasker family, a large majority of the sense and antisense reads mapped to either L1 elements or an “Unknown” family lacking a RepeatMasker family annotation. For the sense reads, mapping to the L1 family and the “Unknown” category (comprising mainly 5S RNA, small cytoplasmic RNA (scRNA), and LSU-rRNA_Hsa) dominates the other families. Notably, 131,177 reads in the L1-active and 76,825 reads in the L1-silenced libraries mapped to active L1 elements compared to 1103 and 1345 reads, respectively, against the inactive LINE L2 family (Figure 5A). The high proportion of repeat RNAs expressed against L1 elements is not unexpected as L1 is the only known active retrotransposon in the human genome and, as expected, L1 expression was markedly repressed in the L1-silenced cells. The most interesting finding for the “Unknown” group of the sense reads is the marked increase in reads mapping to the HY1, HY3, HY4 and HY5 categories of scRNA in the L1-silenced cells (with fold changes ranging from 2.6x to 4.0 x), and the marked increase in reads mapping to tRNA and the U3, U8 and U17 snoRNA (small nucleolar RNA) species (Figure 5B). A similar pattern was also seen for the antisense reads except that the “Unknown” group was much smaller. There was an increase in the numbers of antisense reads mapping to L2 elements (but still < 10% of the values for L1) and MIR elements in the L1-silenced cells (Figure 5C). The up-regulation of scRNA and snoRNA expression in the L1-silenced cells is consistent with a generalized up-regulation of rRNA biogenesis and protein synthesis as the L1-silenced cells assume a more differentiated phenotype.

**Figure 6:**
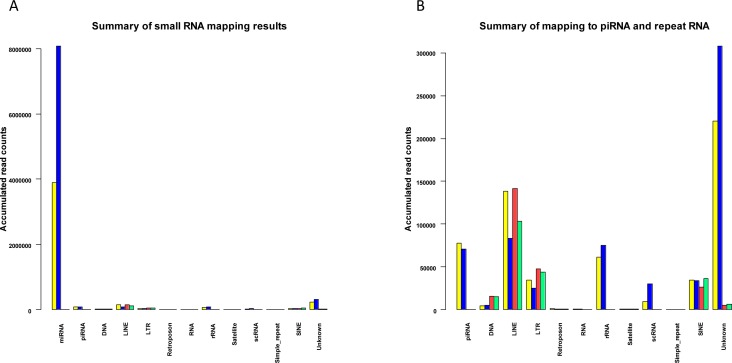
Summary of mapped small RNAs for L1-active and L1-silenced libraries (a) Barplot showing an overview of the accumulated normalized read counts after mapping L1-active and L1-silenced libraries against miRBase, human deepBase sense and antisense rasRNA.fasta and the human piRNABank database. Colour bars: For reads mapped to miRBase and piRNABank: L1-active reads, yellow; L1-silenced reads, blue. For reads mapped to deepBase: L1-active sense reads, yellow; L1-silenced sense reads, blue; L1-active antisense reads, red; L1-silenced antisense reads, green. (b) Higher magnification view of Fig6a after omitting reads mapped to miRBase.

A summary of the small RNA mapping results for the L1 active and L1-silenced libraries is given in Figure 6A-B. As shown in Figure 6A, the expression of piRNAs and repeat-associated small RNA families is at a relatively low level compared to miRNA expression. In mammals, repeat-associated small RNAs have not yet been investigated thoroughly. However, some of the endo-siRNAs that are known to originate from retrotransposons in germline cells have also been shown to be expressed in somatic cells of *Drosophila* and their biogenesis is at least in part, Dicer-dependent [43]. Thus it is plausible that some human repeat-associated small RNAs might exist as remnant copies from germline and embryo cells. At present it remains unclear whether they act as functional siRNAs in controlling repetitive elements in humans, similar to the action of *Drosophila* endo-siRNAs in somatic cells [6]. Because our knowledge of the functional and mechanistic roles of repeat-associated small RNAs is at an early stage, further studies focusing on the regulatory mechanisms of repeat RNA expression will shed light on these questions. Nonetheless, our studies have identified that in addition to miRNAs and piRNAs, repeat RNAs are indeed expressed in breast cancer cells and some of the repeat RNAs identified could potentially regulate the expression of LINEs and other retrotransposons in the human genome.

## DISCUSSION

In light of the accumulating data reporting a connection between the expression of L1 elements and the generation of retrotransposon-derived small RNAs, we analyzed changes in the expression profile of miRNAs, piRNAs and repeat-associated small RNAs before and after silencing the expression of L1 elements in breast cancer cells, using the published L1-specific endo453 sequence that specifically silences endogenous L1 expression [20]. We found that rather than generating small RNAs, L1 expression globally reduces the expression of small RNAs. The principal finding was of greatly increased expression of members of the *let-7* family of miRNAs, and increased expression (above a GFOLD threshold of 2.0) of several other miRNAs including hsa-miR-196a, hsa-miR-30a/d, hsa-miR-191 and hsa-miR-200c following silencing of L1 expression. We also observed increased expression of a number of specific piRNAs, but overall, a decrease in total piRNA expression in the L1-silenced cells. The most highly up-regulated piRNAs were hsa_piR_000586, hsa_piR_001101 and hsa_piR_020450. Remarkably, these piRNA sequences showed a perfect match to C/D box snoRNAs residing in the introns of protein-coding genes, suggesting that expression of such piRNAs may influence gene expression.

Considering the repeat-associated small RNAs that mapped to repetitive elements, in general we found no significant differences in the levels of the repeat RNAs in the presence or absence of L1 expression except for a few antisense RNAs potentially targeting subclasses of retrotransposons. The antisense repeat RNAs that were most differentially expressed between the L1-active and L1-silenced cells mapped to active L1 elements (L1M5, L1ME3), and the MIRc element. The majority of the repeat RNAs analyzed in this study are 18-23 nt in length suggesting that they are endogenous siRNAs as opposed to longer piRNAs, as would be expected in somatic cells. Since the mechanisms of biogenesis of small RNAs derived from repeat elements are not yet known, it is difficult to say whether these repeat RNAs are functional siRNAs, and answering this question would require a functional analysis. The alternative possibility is that leaky transcription from genomic repeats could produce a range of fragmented small RNA sequences, which may or may not be involved in the control of repetitive elements. It is also unclear why the genome expresses sense and antisense repeat RNAs against the oldest and inactive L2 elements. It seems more likely that the L2-derived repeat RNAs are an exaptation of L2 sequences rather than a defense against them. Interestingly, our study shows that most of the repeat-associated small RNA reads mapped to the L1 family and only a small proportion mapped to SINE and LTR elements. The high proportion of repeat RNAs expressed against L1 elements is not unexpected as L1 is the only known active retrotransposon in the genome.

The expression of L1 elements poses a substantial mutagenic threat to genomic stability. To control L1 expression, cells have developed a variety of silencing mechanisms. Several types of small RNAs, including miRNAs, endo-siRNAs and piRNAs, have evolved as defense mechanisms against L1 activity. However, to date, there has been no report of *bona fide* human miRNAs that can target L1 elements. siRNAs can arise from dsRNA precursors, which are processed by the RNAi machinery to generate functional siRNAs against genomic repeats and retrotransposons [2, 4]. In connection with this, our recent study identified a number of L1-specific siRNAs that can markedly silence endogenous L1 expression [20]. In addition, there are some cases of siRNAs and miRNAs originating from the same repetitive elements. For instance, many members of miR-548 family and siRNAs are derived from inverted-repeats of the MADE1 retrotransposon [23]. piRNAs that repress L1 elements have been mainly found in germlines of a number of organisms but not in human somatic cells. Strikingly, recent reports of the presence of Piwi-class proteins suggest that active piRNAs might possibly exist in human cancer cells [38] and in this study we confirmed the presence of piRNAs in breast cancer cells. Notably, we found a number of differentially expressed piRNAs in L1-silenced cells that might potentially be involved in regulating cellular gene expression.

Recent genome-wide studies have revealed that L1 expression and retrotransposition occurs at a high level in the cancer genome, resulting in large-scale restructuring of the genome [14]. Consistent with this, widespread changes in gene expression have been found due to the expression of L1 elements in cancer cells [21]. Also, computational searches have identified a number of miRNAs and snoRNAs that are flanked by L1 sequences, suggesting that L1 activity could potentially influence the expression of small RNAs [25, 27]. However, it is not clear whether the structural and genetic changes caused by L1 retrotransposition might contribute to changes in small RNA expression or whether L1 elements might act as active promoters for small RNA expression. If this was the case, one would expect to observe a positive correlation between L1 expression and small RNA expression. On the contrary, we found that silencing of L1 expression induces the expression of a number of miRNAs. The differential expression of mature *let-7* miRNAs in L1-active and L1-silenced cells is an intriguing finding; similar to the differential expression of *let-7* seen in normal and cancer cells whereby the expression of *let-7* is post-transcriptionally inhibited in cancer cells [44]. Although at present, it is unclear if there is a functional relationship between the expression of *let-7* and L1 elements, there is evidence indicating that cancer cells, which normally express high levels of L1 elements, have globally reduced levels of small RNAs, and in particular, miRNAs, compared to their normal counterparts [45].

At this stage, the underlying mechanism of the activation of the small RNAs in L1-silenced cells is unknown. One possibility is that silencing of L1 expression substantially reduces the restructuring of the genome and the transcriptome that occurs in cancer. One could therefore argue that the relative expression of other RNAs should change in L1-silenced cells. Strikingly, it has been shown that inhibition of L1-encoded reverse transcriptase in several cancer cell types modulates cell growth and differentiation by affecting global gene expression [21]. Another recent study has proposed that the L1-encoded reverse transcriptase enzyme in cancer cells could actively be involved in reverse-transcription of small RNAs and mRNAs into cDNA resulting in the formation of RNA-DNA duplexes and thus impairing the formation of miRNAs and the expression of cellular genes [46]. Inhibiting the activity of RT by pharmacological inhibitors restores the normal profiles of small RNAs and subsequently affects gene regulatory networks within the context of cellular growth. Another possibility is that DNA methylation and chromatin accessibility to RNA polymerase II has a role in the transcriptional activation or silencing of some of these small RNAs. A recent study in human cancer cells suggested that many genomic loci producing small RNAs are subjected to DNA hypermethylation, resulting in transcriptional repression of small RNA expression [47]. Defects in miRNA expression have also been attributed to the transcriptional repression of promoters [48]. Thus, as occurs with miRNAs, it is likely that other classes of small RNAs might also be subjected to DNA methylation-associated repression. It would be interesting to further investigate possible changes in methylation patterns in the promoters of the identified small RNA loci, which might allow us to better understand the biological functions of differentially expressed small RNAs in the human genome. Nevertheless, the data presented in this study suggest that unanticipated interactions exist between the repression of L1 elements and the global expression of small RNAs. Although the exact regulatory pathways are unclear, this study proposes that, through direct silencing of L1 elements, the expression of small RNAs, including miRNAs and piRNAs, plays an important function in the maintenance of genomic integrity.

## METHODS

### Cells, siRNAs and transfection

T47D (ATCC HTB-133) human breast cancer cells were maintained in Dulbecco's modified Eagle's medium (DMEM) with 2 mM L-glutamine and 10% FCS under standard cell culture conditions. An shRNA that mimicked 22-nt of the L1-specific endo453 and a scrambled control shRNA were synthesized as described in reference [49] and cloned directly into a pSM2 vector (Open Biosystems) under the control of a U6 promoter. T47D cells were stably transfected with the scrambled control or L1-specific shRNA constructs using an Amaxa nucleofector kit, followed by puromycin (0.5 μg/ml) selection for 10 days. Depletion of L1 mRNAs was confirmed by qRT-PCR as described previously [20]. Whole cell lysates were prepared by using MPER reagent (Pierce), following the manufacturer's instructions. Western blot analysis was performed with anti-L1-ORF1p antibodies [15] at 1: 2000 dilution, followed by addition of HRP-conjugated secondary antibodies (Silenus, Australia). The resulting signals were visualized using the ECL chemiluminescence system (Pierce). To confirm protein normalization, the membranes were stripped and re-probed with α-tubulin antibodies (Sigma).

### Small RNA library preparation and sequencing

Total RNA from control and endo453-transfected cells was extracted using TRIzol (Invitrogen). Low MW small RNA was enriched by adding 50% PEG-8000 and 5M NaCl to a final concentration of 5% and 0.5M, respectively, followed by gel purification in a 15% urea-PAGE gel (Invitrogen). Small RNAs ranging from 18-30 nt were gel purified and ligated to 3' adaptor and 5' adaptor oligonucleotides as described in the Illumina Small RNA Kit. Briefly, the 3'-RNA adaptor (5'/5rApp/ATCTCGTATGCCGTCTTCTGCTTG/3ddc/), which specifically ligates to RNAs that contain a hydroxyl group at their 3'end, was ligated to 1 μg of low MW small RNAs using T4 RNA ligase (NEB). The resulting products were subsequently ligated to the 5'-RNA adaptor (5'-GUUCAGAGUUCUACAGUCCGACGAUC-3'). cDNA was synthesized with SuperScript II Reverse Transcriptase (Invitrogen) and subjected to 12 cycles of PCR amplification with high-fidelity Phusion Polymerase (NEB) using primers as published by Illumina. Each library was loaded on a single Illumina lane at 20 pM and underwent 36 cycles of sequencing on an Illumina GAIIx Genome Analyzer.

### Bioinformatic analysis of small RNAs

Singleton reads and reads with a 3'-adapter substring <6 nt or trimmed sequence length <17 nt were removed from the analysis carried out by the miRanalyzer pipeline. Trimmed reads were aligned to the miRBase database of human miRNAs (version 16) using Bowtie as implemented in the miRanalyzer pipeline (with the following Bowtie options: minimum score of 30 and minimum identity of 90%) [31]. Many miRNA sequences, especially those belonging to the same miRNA family, have a high degree of sequence similarity and since the read may be short (~ 16 bp), non-unique matches can occur. A non-unique match exists if a read maps with the same quality (i.e. same number of mismatches) to different positions or to more than one sequence in the library. Failure to report non-unique matches may represent loss of information. Therefore, miRanalyzer reports ambiguous matches, listing all miRNAs where matches have been found. The order of mapping against known miRNAs is firstly to mature, then mature-star and precursors/hairpin. Both unique matches (a read matching to a single known microRNA) and ambiguous matches are detected and removed from the input at each step. The sequential removal is important as otherwise the reads would be detected again in the precursor sequences (hairpins). For further details, refer to: http://bioinfo2.ugr.es/miRanalyzer/miRanalyzer_tutorial.html#x1-110004.1.1 In order to calculate differential expression, the mapped read counts were normalized and fold changes between L1-active and L1-silenced cells were calculated with the R/Bioconductor DESeq package [32] which is integrated into the miRanalyzer pipeline.

### Detection and analysis of piRNAs and repeat-associated RNAs

To identify differentially expressed piRNAs, we selected the trimmed reads in each library with lengths greater than 23 nucleotides using the filter function in CLC Genomics Workbench 5.5.1 and mapped these subsets of reads against the human piRNABank database [30] using CLC Genomics Workbench with default parameters (i.e. maximum mismatches of 2 and strand-specific alignment). Each file of adapter-trimmed Illumina reads was aligned to the uncompressed Human.tar.gz piRNABank fasta file after converting the fasta reads from RNA to DNA. The two files of mapped reads and read counts (for the L1-active and L1-silenced libraries) were exported in plain text format from CLC Genomics Workbench and merged in R using the merge() command. The read counts were normalized using the DESeq package in R.

To identify differentially expressed RNAs mapping to repetitive elements, we selected the trimmed reads in each library with lengths less than 23 nucleotides using the filter function in CLC Genomics Workbench. Repeat RNAs were identified and annotated by alignment with the known rasRNA sequences in the deepBase database (version GRCh37)[31]. Alignments were carried out with CLC Genomics Workbench using default parameters (i.e. maximum mismatches of 2 and strand-specific alignment). Each subset of adapter-trimmed Illumina reads was aligned to the Human_sense_rasRNA_deepBase.fasta and Human_antisense_ rasRNA_deepBase.fasta files. The two files containing the mapped reads from the L1-active and L1-silenced libraries were exported from CLC Genomics Workbench and edited in MS Excel to remove extraneous text strings before importing and merging in R with the merge() command. The read counts were normalized using the DESeq package in R. The fasta headers of each sequence (containing the deepBase accession name, genomic coordinates and RepeatMasker name) from the human deepBase.fasta files were extracted and saved to a text file using the Linux grep command. The file containing the normalized reads from both libraries was merged with the file of fasta headers using the R merge() command. The RepeatMasker class and family for each RepeatMasker name were downloaded into a text file from the RepeatMasker [42] website with a Perl script and further processed in MS Excel to remove extraneous columns and html tags before adding the RepeatMasker class and family names to the normalized reads file using the R merge() command. Read counts were accumulated for RepeatMasker classes and families using R. Additional details of the bioinformatics workflow and the Perl and R scripts are available on request.

## SUPPLEMENTARY TABLES


